# 1934. Nirsevimab is Associated with Higher and More Sustained RSV Neutralizing Antibody Responses Compared with Standard of Care Palivizumab: Observations from a 2:1 Randomized, Phase 2/3 Trial in Medically Vulnerable Children (MEDLEY)

**DOI:** 10.1093/ofid/ofad500.2465

**Published:** 2023-11-27

**Authors:** Deidre Wilkins, Ulrika Wählby Hamrén, Yue Chang, Joseph B Domachowske, Janet A Englund, William J Muller, Amanda Leach, Tonya L Villafana, Elizabeth J Kelly

**Affiliations:** Translational Medicine, Vaccines & Immune Therapies, BioPharmaceuticals R&D, AstraZeneca, Gaithersburg, MD; AstraZeneca, Gothenburg, Sweden, Mölndal, Vastra Gotaland, Sweden; AstraZeneca, Gaithersburg, Maryland; SUNY Upstate Medical University, Syracuse, New York; Seattle Children’s Hospital, Seattle, Washington; Ann and Robert H. Lurie Children’s Hospital of Chicago and Northwestern University Feinberg School of Medicine, Chicago, Illinois; AstraZeneca, Gaithersburg, Maryland; AstraZeneca, Gaithersburg, Maryland; AstraZeneca, Gaithersburg, Maryland

## Abstract

**Background:**

Nirsevimab is an extended half-life monoclonal antibody (mAb) newly authorized for the prevention of respiratory syncytial virus (RSV) lower respiratory tract disease in infants born during or entering their first RSV season, and in children up to 24 months of age who remain vulnerable to severe disease through their second season. Anti-RSV mAbs are currently assessed based on their direct virus neutralization activity in the absence of a well-established correlate of protection. We explored trends in anti-RSV neutralizing antibody (nAb) levels in participants dosed with nirsevimab, or palivizumab, the standard of care during the MEDLEY trial (NCT03959488).

**Methods:**

nAb levels following dosing with nirsevimab or palivizumab were evaluated in infants born preterm (for season [S] 1) or with chronic lung disease or congenital heart disease (CLD/CHD) across two RSV seasons (S1/2) **(Fig. 1)**. Participants provided blood samples at baseline and during site visits on Day (D) 31, 151, and 361 of their respective seasons. nAb levels were determined using a fluorescent focus-based microneutralization assay. Pharmacokinetic (PK) modeling was used to predict peak and trough palivizumab nAb levels.

**Figure 1.**
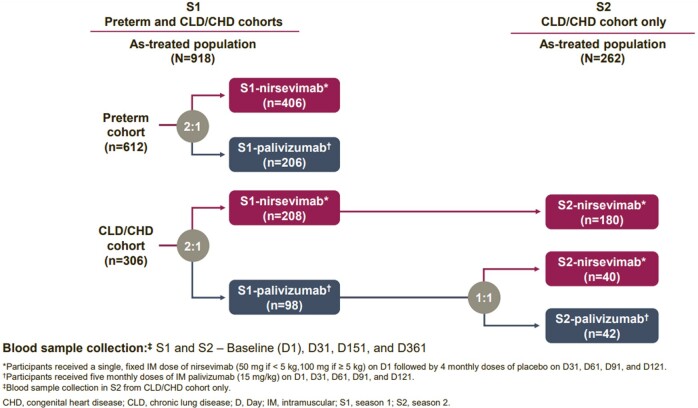
MEDLEY trial design and number of participants per cohort

**Results:**

The dynamics of nAb levels during the trial are depicted in **Fig 2.** As expected, nAb levels in S1-nirsevimab recipients were highest in samples collected during the first visit, D31, and declined over S1, but were still > 16-fold above baseline at D361 **(Fig. 2A)**. nAb levels in S1-palivizumab recipients increased incrementally and were highest in samples obtained at the D151 visit. nAb levels at D151 were ∼10-fold higher with S1-nirsevimab compared with S1-palivizumab. S1 D361 (baseline S2) nAb levels among participants who received palivizumab in S1 fell towards the assay’s lower limit of detection **(Fig. 2A+B)**. Overall patterns in nAb levels in S2 were similar to those in S1. S1-palivizumab/S2-palivizumab recipients had ∼10-fold lower levels of nAbs over the S2 PK model-predicted time course to D151 compared with participants receiving S2-nirsevimab.

**Figure 2.**
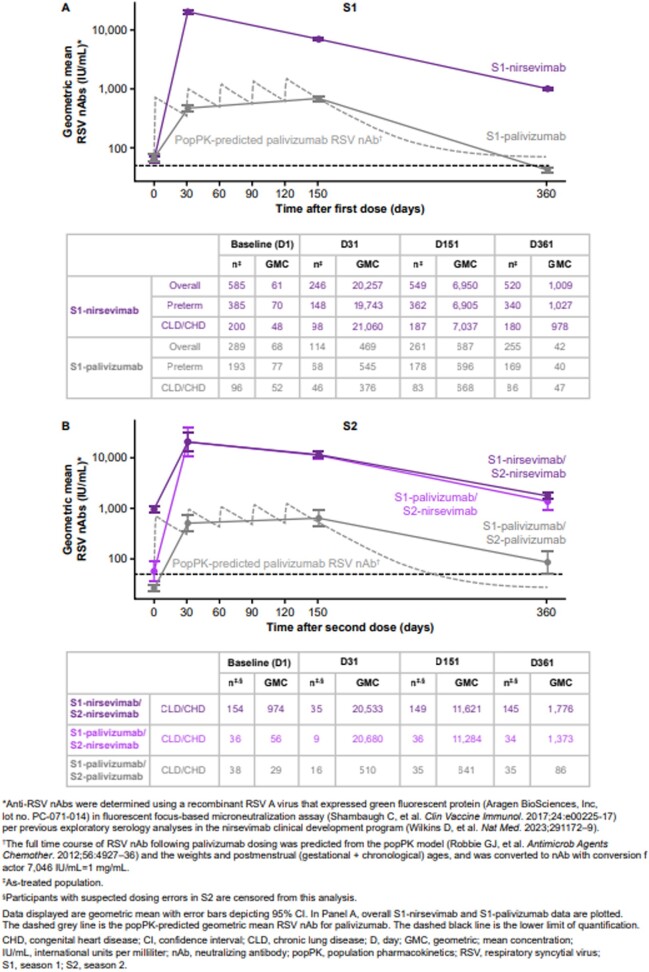
Comparison of RSV nAb levels among MEDLEY participants across S1 and S2

**Conclusion:**

Nirsevimab is associated with ∼10-fold higher and sustained levels of nAbs through 1 year post dose compared with palivizumab, suggesting nirsevimab may offer protection for a period beyond a typical 150-day RSV season.

**Disclosures:**

**Deidre Wilkins, BSC**, AstraZeneca: Employee|AstraZeneca: Stocks/Bonds **Ulrika Wählby Hamrén, PhD**, AstraZeneca: Patent holder re. dosing|AstraZeneca: Employee|AstraZeneca: Stocks/Bonds **Yue Chang, PhD**, AstraZeneca: Employee|AstraZeneca: Stocks/Bonds **Joseph B. Domachowske, MD**, AstraZeneca: Advisor/Consultant|AstraZeneca: Grant/Research Support|GlaxoSmithKline: Advisor/Consultant|GlaxoSmithKline: Grant/Research Support|GlaxoSmithKline: Honoraria|Merck: Grant/Research Support|Moderna: Grant/Research Support|Pfizer: Grant/Research Support|Sanofi: Advisor/Consultant|Sanofi: Grant/Research Support **Janet A. Englund, MD**, AbbVie: Advisor/Consultant|Ark Biopharmaceutical: Advisor/Consultant|AstraZeneca: Advisor/Consultant|AstraZeneca: Grant/Research Support|GlaxoSmithKline: Advisor/Consultant|GlaxoSmithKline: Grant/Research Support|Meissa Vaccines: Advisor/Consultant|Meissa Vaccines: Grant/Research Support|Merck: Grant/Research Support|Pfizer: Advisor/Consultant|Pfizer: Grant/Research Support|Sanofi Pasteur: Advisor/Consultant **William J. Muller, MD**, Ansun Biopharma: Grant/Research Support|Astellas Pharma: Grant/Research Support|AstraZeneca: Advisor/Consultant|AstraZeneca: Grant/Research Support|DiaSorin Molecular LLC: Advisor/Consultant|Eli Lilly and Company: Grant/Research Support|Enanta Pharmaceuticals: Grant/Research Support|F. Hoffmann-La Roche: Grant/Research Support|Finley Law Firm, P.C.: Expert Testimony|Gilead Sciences: Grant/Research Support|Invivyd: Advisor/Consultant|Janssen Biotech: Grant/Research Support|Karius, Inc.: Grant/Research Support|Melinta Therapeutics, Inc.: Grant/Research Support|Merck: Grant/Research Support|Moderna: Grant/Research Support|Nabriva Therapeutics plc: Grant/Research Support|Paratek Pharmaceuticals, Inc.: Grant/Research Support|Pfizer: Grant/Research Support|Sanofi Pasteur LLC: Advisor/Consultant|Tetraphase Pharmaceuticals, Inc.: Grant/Research Support **Amanda Leach, MD**, AstraZeneca: Employee|AstraZeneca: Stocks/Bonds **Tonya L. Villafana, PhD, MPH**, AstraZeneca: Employee|AstraZeneca: Stocks/Bonds **Elizabeth J. Kelly, PhD**, AstraZeneca: Employee|AstraZeneca: Stocks/Bonds

